# Melatonin Supplementation Enhances Next-Day High-Intensity Exercise Performance and Recovery in Trained Males: A Placebo-Controlled Crossover Study

**DOI:** 10.3390/sports13060190

**Published:** 2025-06-19

**Authors:** Nourhène Mahdi, Slaheddine Delleli, Arwa Jebabli, Khouloud Ben Maaoui, Juan Del Coso, Hamdi Chtourou, Luca Paolo Ardigò, Ibrahim Ouergui

**Affiliations:** 1 High Institute of Sport and Physical Education of Sfax, University of Sfax, Sfax 3000, Tunisia; nourhene648@gmail.com (N.M.); sdelleli2018@gmail.com (S.D.); jebabliarwa@gmail.com (A.J.); benmaaouikhouloud88@gmail.com (K.B.M.); h_chtourou@yahoo.fr (H.C.); 2 Research Laboratory, Education, Motricity, Sport and Health, EM2S, LR19JS01, University of Sfax, Sfax 3000, Tunisia; 3 Physical Activity, Sport and Health, Research Unit, UR18JS01, National Sport Observatory, Tunis 1003, Tunisia; 4 Centre for Sport Studies, Rey Juan Carlos University, 28943 Fuenlabrada, Spain; juan.delcoso@urjc.es; 5 Department of Teacher Education, NLA University College, 0166 Oslo, Norway; 6 High Institute of Sport and Physical Education of Kef, University of Jendouba, Kef 7100, Tunisia; 7 Research Unit: Sport Sciences, Health and Movement, UR22JS01, University of Jendouba, Kef 7100, Tunisia

**Keywords:** ergogenic aid, muscle damage, inflammation, recovery, sleep, sports performance

## Abstract

Background/Objectives: Sleep and recovery are critical for optimising exercise performance. However, the efficacy of melatonin supplementation in improving sleep quality and next-day physical performance remains unclear. This study examined the effects of melatonin ingestion on sleep and performance-related outcomes the following day in trained males. Methods: In a randomised, double-blind, placebo-controlled crossover study, 12 trained males (age: 21.92 ± 2.84 years) ingested 6 mg of melatonin (MEL) or a placebo (PLA) the night before performing the 5 m shuttle test (5mSRT). Before and after the 5mSRT, blood samples were collected. Peak heart rate (HR_peak_) and rating of perceived exertion (RPE) were recorded throughout the test. Perceived recovery status (PRS) and delayed onset muscle soreness (DOMS) were measured before, 5 min, 24 h, 48 h, and 72 h after the test. The sleep/wake cycle was monitored during the night after ingestion. Results: Data were analysed using paired t-tests, Wilcoxon tests, and two-way ANOVAs, with significance set at *p* < 0.05. Compared to PLA, MEL did not modify any sleep parameters or blood markers (all *p* > 0.05). However, MEL improved total distance, fatigue index, the percentage decrement between sprints, and HR_peak_ (all *p* < 0.05) in the 5mSRT compared to PLA. MEL also enhanced PRS values up to 72 h post-exercise and reduced DOMS (all *p* < 0.05). Conclusion: In summary, 6 mg of melatonin taken at night enhanced next-day high-intensity exercise performance and improved perceived recovery up to 72 h post-exercise.

## 1. Introduction

It is well-documented that intense physical exercise can induce inflammation and cause skeletal muscle damage, initiating processes essential for muscle repair and adaptation [1,2]. Various recovery strategies, including antioxidant supplementation, have been used as ways to mitigate the effects of muscle damage and inflammation caused by intense physical exercise [3,4]. Antioxidants are considered promising dietary supplements for counteracting exercise-induced muscle damage by reducing the production of oxidative stress and modulating inflammation [5]. Melatonin (MEL) (N-acetyl-5-methoxytryptamine) has been identified as an antioxidant with the potential to promote recovery after intense exercise [6], and enhance physical performance in subsequent exercise sessions [3]. MEL, often referred to as a chronobiotic, is a hormone synthesized by the pineal gland [7,8] and is well known for regulating biological rhythms in mammals [9]. The synthesis and secretion of MEL are primarily regulated by the light–dark cycle, being triggered during the transition to darkness and strongly inhibited by exposure to light, particularly blue wavelengths [9,10]. MEL is also recognized for its protective role in safeguarding mitochondria from damage caused by reactive oxygen species (ROS) and reactive nitrogen species (RNS), acting as a potent scavenger and reducing oxidative and nitrosative stress [11]. This protective effect may help reduce muscle fatigue, as oxidative stress is a known limiting factor in athletic performance [10]. Given that athletic performance is closely linked to sleep patterns and quality [12,13], exogenous MEL supplementation has been investigated as a strategy to enhance sleep quality [14,15], ultimately improving physical and mental recovery after exercise [16]. However, the literature on the potential benefits of exogenous MEL intake is scarce [17], and to the best of our current knowledge, only a few studies have investigated the effect of MEL ingestion before bedtime on exercise performance the following day [2,18,19].

In healthy adolescents, 10 mg of MEL ingested in the evening after performing Yo-Yo Intermittent Recovery Test Level 1 (YYIRT-1) improved the quality and quantity of sleep and enhanced some physical performance parameters the following morning [18]. Similarly, Cheikh et al. [2] reported that the ingestion of 10 mg of MEL the night before significantly improved biomarkers of cellular damage and inflammation induced by the Running-Based Anaerobic Sprint Test (RAST) in healthy adolescents. Last, Ghattassi et al. [19] reported that a nocturnal dose of 5 mg of MEL improved subjective sleep quality and enhanced performance in the Wingate anaerobic test and other short-term performance assessments in professional soccer players. However, the optimal dose of MEL for maximizing sleep and exercise performance benefits remains unclear, as does the duration of its effects, whether they persist for several hours or even days after an intense exercise session. A dose within the 5 to 10 mg range may represent a reasonable compromise between efficacy and tolerability; however, to the authors’ knowledge, no other studies have examined melatonin within this dosage range specifically in the context of intense exercise and follow-up assessments extending several days post-exercise.

This study aimed to assess the effects of acute nighttime oral MEL administration on sleep parameters, physical performance, and biochemical markers of muscle damage and inflammation following a short, repetitive maximal exercise test performed the following day, with follow-up assessments extending up to 72 h post-exercise. Based on the existing literature [2,18,19], we hypothesized that ingesting a moderate dose of 6 mg of melatonin at night will improve sleep quality and duration, reduce markers of muscle damage and inflammation, and enhance exercise performance the following morning, with beneficial effects on perceived recovery and muscle soreness persisting for up to 72 h post-exercise.

## 2. Materials and Methods

### 2.1. Participants

**Figure 1:**
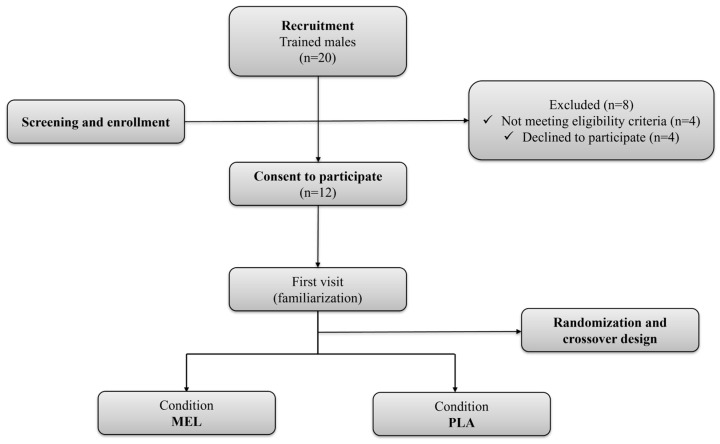
Flowchart of participants. MEL: melatonin; PLA: placebo.

The G*Power software (version 3.1.9.4, University of Kiel, Kiel, Germany) was used to estimate the sample size, using the F test family (repeated measures, within factors) as the statistical method of analysis, with two conditions and one group of participants. Based on the performance effects of MEL in previous studies [18,20], the required sample size was 8 participants (effect size f = 0.6 (medium), α = 0.05) with a statistical power of 80%. To be eligible, participants should be (i) trained athletes (≥3 h of exercise training/week), (ii) non-smoking individuals with no significant medical restrictions, (iii) not using dietary supplements or anti-inflammatory drugs, (iv) not completed trans-meridian travels in the month before the study, (v) have not experienced sleep disorders in the last two months. From an initial pool of 20 potential participants, eight were deemed ineligible either for failing to meet one or more inclusion [21] criteria or for declining participation after a detailed explanation of the experimental procedures. Therefore, the final sample was composed of 12 trained males (Table 1). Overall, the participants were team sport athletes or short-term individual sport athletes who engaged in high-intensity intermittent exercise 1–2 times per week and were familiar with shuttle running tests. Based on their responses to the Horne and Ostberg self-assessment questionnaire [22], participants were categorized as either ‘moderately morning type’ (*n* = 5) or ‘neither type’ (*n* = 7). All participants gave written informed consent after being informed about a detailed explanation of the study procedures, including potential risks and possible discomforts. The study was conducted according to the Declaration of Helsinki and was approved by a local research ethical committee (CCP N°0411/2022) before the commencement of the experiment. The flow diagram of participants’ recruitment and participation is shown in Figure 1.

### 2.2. Experimental Design

**Figure 2:**
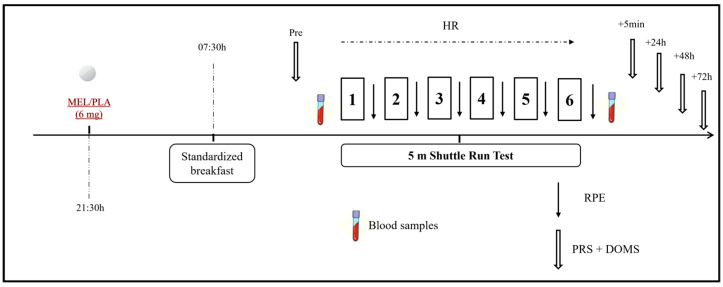
Schematic representation of the study design. MEL: melatonin; PLA: placebo; RPE: rating of perceived exertion; PRS: perceived recovery scale; HR: heart rate.

This was a randomized double-blind placebo-controlled crossover study in which physical, biochemical, physiological, and perceptual responses as well as sleep assessment were performed under the intake of 6 mg of MEL (vegetable source made by Webber Naturals, Belowna, BC, Canada) and placebo (PLA; composed of lactose, starch, and cellulose) (Figure 2). The dose of MEL was determined based on previous studies [4,12], ensuring absorption through the mucosal membrane and falling within the range recommended for human use (i.e., 3–20 mg) [23]. One week before the commencement of the experiment, participants underwent a familiarization session that included all the experimental procedures (excluding blood sampling) as they would be performed in the experimental trials. Assessments were performed in two separate experimental trials (MEL and PLA) with a one-week interval to allow sufficient washout and recovery between conditions [2]. Experimental trials were identical except for the substance ingested. Performance measurements were performed at the same time of day (09:00–10:00) to avoid any diurnal variation in the variables under investigation. The order randomization of the trials was carried out by an independent staff member who was not involved in the measurement process. Additionally, participants were instructed and supervised to avoid discussing or comparing tastes or making assumptions about their ingestion. During the 24 h prior to each experimental session, participants were instructed to avoid strenuous exercise and followed generalized fluid and diet guidelines to ensure appropriate energy balance and hydration before all experimental trials [24]. In addition, participants were required to abstain from alcohol, caffeine, and other stimulants consumption 72 h before each trial. To assure a within-subject diet standardization, participants were requested to complete a dietary record in the 24 h leading up to the first trial. Then, this dietary pattern was replicated before the second trial.

Each experimental condition lasted for two days (day 1 and day 2). On Day 1, participants followed their regular daily routines without engaging in any exercise. Around 30 min before sleep onset (at 21:30), participants ingested the MEL or PLA [25] in a capsule of the same appearance for both substances with 100 mL of water. Then, they went to sleep and sleep parameters were monitored during the night using an ActiGraph GT3X placed on the non-dominant arm (ActiGraph, Pensacola, FL, USA) following the protocol described elsewhere [26].

On the following morning (Day 2), participants were awakened at 07:00 and they had a standardized breakfast at 07:30 min. To ensure consistency in morning procedures and avoid potential variations associated with different waking times, all participants were awakened at the same time. Sleep diaries and ActiGraph recordings confirmed that no participant awoke before the scheduled time. After 2 h to allow digestion, they performed the 5 m shuttle test (5mSRT), as described below and following the protocols used in prior investigations [23,27]. Before and after the test, venous blood samples were taken to determine concentrations of muscle damage and inflammation markers, as described below. Heart rate (HR) was continuously measured during the exercise test with a frequency of 1 Hz (Polar team2 Pro System, Polar Electro OY, Kempele, Finland), and peak heart rate (HRpeak) was obtained as the highest heart rate value obtained during the test. The rating of perceived exertion (RPE) was recorded immediately after each repetition of the 5mSRT using the Borg CR-10 scale [28] and the scores obtained were summed over the number of repetitions. Scores for self-perceived recovery (PRS) [29] and delayed onset muscle soreness (DOMS) [30] were assessed at multiple time points: before, 5 min, 24 h, 48 h, and 72 h post-exercise.

### 2.3. Data Collection and Analysis

#### 2.3.1. 5 m Shuttle Run Test (5mSRT)

The 5mSRT is a fitness assessment test used to measure agility, speed, and anaerobic capacity [31]. For this test, participants ran back and forth over a 5 m distance for a 30 s period for a total number of 6 repetitions [32]. In all the repetitions, participants had to cover the greatest possible distance, and the total distance in each repetition was measured from the starting point to the point where the participant crossed the line. If he finished the time and was between two lines without fully crossing the second line (for example, halfway between the 10 m and 15 m lines), the distance covered was considered incomplete. The measurement was taken based on the last line they fully crossed before the time expired. A 35 s recovery period was allowed between repetitions. Two lanes of 1 m in width and placed 5 m apart were used to mark the place where participants had to change the direction of running [32]. The following parameters were measured in each 5mSRT:

- Peak distance (HD) (m): the greatest distance achieved in any single 30 s shuttle sprint [32]. Total distance (TD) (m): the cumulative distance covered across all six 30 s shuttle runs [32].
- Fatigue index (FI) (%) = {[((Shuttle 1 + Shuttle 2)/2) − ((Shuttle 5 + Shuttle 6)/2)]/((Shuttle 1 + Shuttle 2)/2)} × 100 [33].
- Percentage decrement (PD) (%) = [((HD × number of sprints) − TD))/(HD × number of sprints)] × 100 [34].

#### 2.3.2. Rating of Perceived Exertion

Perceived exertion was determined using the Borg CR-10 scale [28]. This scale ranges from 0 to 10, with corresponding verbal expressions in which the intensity of the perceived sensation increases progressively (i.e., 0 = nothing at all and 10 = extremely strong). The mean of the scores recorded after each 5mSRT repetition was used for the statistical analysis and obtained through the following formula [35]:

RPE (a.u) = Σ (RPE scores)/number of repetitions

#### 2.3.3. Perceived Recovery Scale

Participants determined their perceived recovery using an 11-point scale where a score of “0” indicated very little recovery or extremely tired and “10” represented very good recovery or very energetic [29].

#### 2.3.4. Delayed Onset Muscle Soreness

DOMS was determined using a 10-point scale ranging from ‘1’ (no pain) to ‘10’ (very, very painful) which reflects a subjective assessment of muscle pain in the lower limbs [30].

#### 2.3.5. Blood Analyses

Venous blood samples were collected from an antecubital vein using serum/plasma separator tubes following 5 min of sitting rest (pre-test) and again 5 min after completing the 5mSRT. The samples were analyzed for biomarkers including creatine kinase (CK), lactate dehydrogenase (LDH), aspartate aminotransferase (ASAT), alanine aminotransferase (ALAT), and C-reactive protein (CRP). A heparinized tube was used to determine CK, LDH, ASAT, and ALAT concentrations, while CRP concentration was measured from a tube without anticoagulant. All biochemical assays were carried out using standard techniques at the laboratory of Kef Regional Hospital, Kef, Tunisia, using the Selectra Pro XL automated machine. The spectrophotometric or kinetic method at 340 nm was used to measure CK, LDH, ASAT, and ALA, and the immunoturbidimetric method for CRP. The intra-assay coefficient of variation (CV) for these parameters was 1.3%, 0.2%, 1.1%, 1.5%, and 1.16%, respectively. Serum/plasma was separated by centrifugation within 15 min of collection at 4000 rpm.

#### 2.3.6. Actigraph Registration and Analysis

In each experimental session, participants wore the GT3X activity monitors during the night, on the wrist of the non-dominant arm (ActiGraph, Pensacola, FL, USA). Sleep/wake cycles were analyzed using ActiLife software (Actilife version 6.13.4), with key variables recorded bedtime, out bedtime, sleep latency, sleep efficiency, total time in bed, and total sleep time.

### 2.4. Statistical Analyses

All statistical analyses were processed using SPSS software version 23.0 (IBM Corp., Armonk, NY, USA). To check and assess the normality of each variable, the Shapiro–Wilk test was applied. Data was presented as mean and standard deviation for variables with a normal distribution, and median and interquartile range for variables with non-normal distribution. Homogeneity of variances was assessed using Levene’s test, while Mauchly’s test was used to evaluate the assumption of sphericity. For data that were normally distributed, paired T-tests were used to compare conditions for bedtime, sleep latency, sleep efficiency, total time in bed, FI, HRpeak, and RPE. Additionally, a two-way analysis of variance (ANOVA 2 × 2) [supplement (MEL and PLA) × time (before and after)] was employed for ASAT and ALAT. When appropriate, the Bonferroni test was used as a post hoc procedure. A partial eta-square (ηp^2^) effect size was computed, and values were classified as small (0.01), moderate (0.06), and large (0.13) [4,36]. The upper and lower 95% confidence intervals of the difference (95% CIs) were calculated for each normally distributed variable. In cases where normality was biased, the Wilcoxon test was used for out-of-bed time, total sleep time, BD, TD, and PD, and a Friedman non-parametric test was used for CRP, LDH, CPK, PRS, and DOMS. When the Friedman test showed a significant difference, the Wilcoxon signed-rank test was used as post hoc. The correlation coefficient (r) was calculated using the Wilcoxon Z-scores and the total number of observations (N) (i.e., r = Z/√N) and considered as follows: 0.1 to <0.3 (small), 0.3 to <0.5 (moderate), and ≥0.5 (large) [37]. Additionally, standardized effect size analysis (Cohen’s d) was used to evaluate the magnitude of differences between variables, and interpreted as follows: trivial (≤0.20); small (≤0.60); moderate (≤1.20); large (≤2.0); very large (≤4.0); and extremely large (>4.0) [38]. Statistical significance was set at *p* < 0.05 for all analyses.

## 3. Results

### 3.1. Sleep Parameters

Statistical analysis showed that all sleep parameters were similar between the two conditions: In bedtime (t = 0.122; *p =* 0.905); out bedtime (Z = −0.353; r = 0.101; *p =* 0.724); sleep latency (t = 0.547; *p =* 0.595; d = 0.065); sleep efficiency (t = −1.323; *p =* 0.213; d = 0.479.); total time in bed (t = −0.477; *p =* 0.643; d = 0.147); total sleep time (Z = −1.844; r = 0.532; *p =* 0.065) (Table 2).

### 3.2. Physical and Physiological Parameters

For best distance, there was no significant difference between the two conditions (Z = −1.386; r = 0.400; *p =* 0.166). However, for total distance, the Wilcoxon test showed higher values after MEL ingestion compared to PLA (Z = −2.824; r = 0.815; *p* < 0.05). Additionally, FI was lower after MEL ingestion compared to PLA (t = 4.776; *p =* 0.001; d = 1.6887) along with a lower percentage decrement after MEL (Z = −2.824; r = 0.815; *p* < 0.05). Regarding HRpeak, values were lower after MEL ingestion compared to PLA (t = 2.462; *p =* 0.032; d = 0.0684). However, RPE values were similar between the two conditions (t = 1.024; *p =* 0.328; d = 0.3991) (Table 3).

### 3.3. Biochemical Parameters

#### 3.3.1. Creatine Kinase (CK)

Statistical analysis revealed a significant time effect (Chi^2^ = 13.8; N = 12; df = 3; *p =* 0.003). Pairwise comparison revealed that CK increased after the 5mSRT in PLA (Z = −3.059; r = 0.883; *p =* 0.002) and MEL (Z = −3.063; r = 0.884; *p =* 0.002) conditions compared to before. However, there were no significant differences between the MEL and PLA conditions before (Z = −0.392; r = 0.113; *p =* 0.695) and after (Z = −0.235; r = 0.067; *p =* 0.814) exercises (Table 4).

#### 3.3.2. Lactate Dehydrogenase

The statistical analysis showed a significant time effect (Chi^2^ = 23.92; N = 12; df = 3; *p* < 0.001). Pairwise comparison showed that serum LDH concentration increased after exercise in both PLA (Z = −3.062; r = 0.883; *p =* 0.002) and MEL (Z = −3.064; r = 0.884; *p =* 0.002) conditions. However, there were no significant differences between the MEL and PLA conditions before (Z = −0.708; r = 0.204; *p =* 0.479) and after exercise (Z = −1.334; r = 0.385; *p =* 0.182) (Table 4).

#### 3.3.3. Aspartate Aminotransferase

There was a main effect of time (F1,11 = 36.52; *p* < 0.001; ηp^2^ = 0.77), with higher values after the 5mSRT than before (95%CI: 2.20 to 4.72; d = 1.01; *p* < 0.001). However, no significant main effect of substance (F1,11 = 0.17; *p =* 0.69; ηp^2^ = 0.015), or interaction effect between time and supplement (F1,11 = 3.40; *p =* 0.09; ηp^2^ = 0.24 (Table 4)

#### 3.3.4. Alanine Aminotransferase

There was a significant main effect of time (F1,11 = 20.29; *p =* 0.001; ηp^2^ = 0.65), with higher values recorded after the 5mSRT than before (95%CI: 1.39 to 4.03; d = 0.94; *p =* 0.001). However, there was no main effect of supplement (F1,11 = 0.46; *p =* 0.51; ηp^2^ = 0.04), or interaction effect between time and supplement (F1,11 = 0.47; *p =* 0.50; ηp^2^ = 0.04) (Table 4).

#### 3.3.5. C-Creative Protein

The Friedman test showed a significant condition effect (Chi^2^ = 9.45; N = 12; df = 3; *p =* 0.024). However, the post hoc analysis did not report any pre-to-post exercise change in any condition nor differences between PLA and MEL at any time point (Table 4).

### 3.4. Perceived Recovery Scale

**Figure 3:**
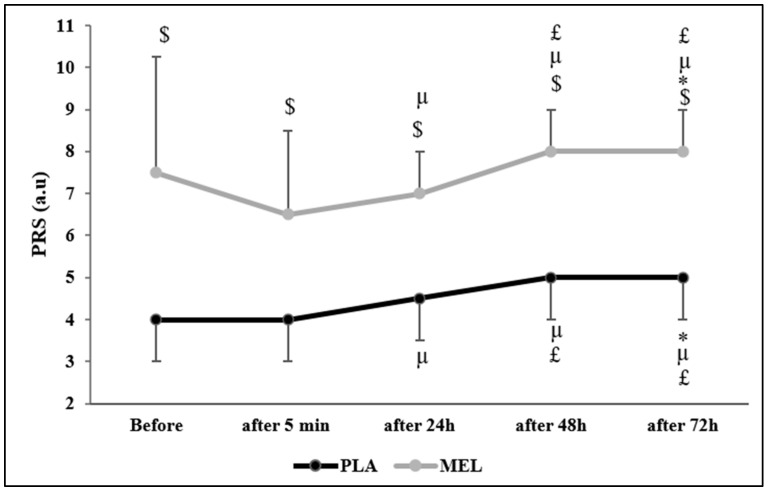
Perceived recovery status (PRS) scores recorded before, 5 min, 24 h, 48 h, and 72 h after the 5 m shuttle run test in PLA and MEL condition (n = 12). Values are presented as median (interquartile range); * *p* < 0.05 significant difference in comparison with values measured before the 5mSRT within each condition. µ *p* < 0.05: significant difference in comparison with values measured 5 min after the 5mSRT within each condition. £ *p* < 0.05: significant difference in comparison with values measured 24 h after the 5mSRT within each condition. $ *p* < 0.05 significant difference between PLA and MEL at time pairwise comparisons. MEL: melatonin; PLA: placebo; a.u: arbitrary unit.

The Friedman test reported a significant effect of time on PRS values across conditions (Chi^2^ = 75.553; N = 12; df = 9; *p* < 0.001). The Wilcoxon test showed a significant effect within the PLA condition, with PLA before the 5mSRT resulting in lower values than PLA 72 h after (Z = −2.164; r = 0.624; *p* = 0.030) and MEL before (Z = −2.956; r = 0.853; *p =* 0.003). Moreover, PLA elicited lower values 5 min after the test than PLA 24 h (Z = −2.236; r = 0.654; *p* = 0.025), 48 h (Z = −2.599; r = 0.750; *p =* 0.009), 72 h (Z = −2.537; r = 0.732; *p =* 0.011), and MEL 5 min (Z = −2.829; r = 0.816; *p =* 0.005) after exercise. Furthermore, PLA 24 h after exercise elicited lower values than PLA 48 h (Z = −2.449; r = 0.706; *p =* 0.014), 72 h (Z = −2.373; r = 0.685; *p =* 0.018), and MEL 24 h (Z = −2.816; r = 0.812; *p =* 0.005) post-exercise. In the MEL condition, MEL 5 min after exercise elicited lower values than MEL after 24 h (Z = −2.236; r = 0.645; *p =* 0.025), 48 h (Z = −2.460; r = 0.710; *p* = 0.014), and 72 h (Z = −2.980; r = 0.860; *p =* 0.003). Additionally, MEL 24 h after the 5mSRT elicited lower values than MEL after 48 h (Z = −2.333; r = 0.673; *p =* 0.020). MEL after 48 h also resulted in lower values than PLA 48 h (Z = −2.831; r = 0.817; *p =* 0.005). Lastly, MEL 72 h post-exercise showed significantly higher values compared to MEL after 48 h (Z = −2.828; r = 0.816; *p* = 0.005), 24 h (Z = −3.066; r = 0.885; *p =* 0.002), 5 min (Z = −2.980; r = 0.860; *p =* 0.003), and MEL before testing (Z = −2.106; r = 0.607; *p =* 0.035) (Figure 3).

### 3.5. Delayed Onset Muscle Soreness

**Figure 4:**
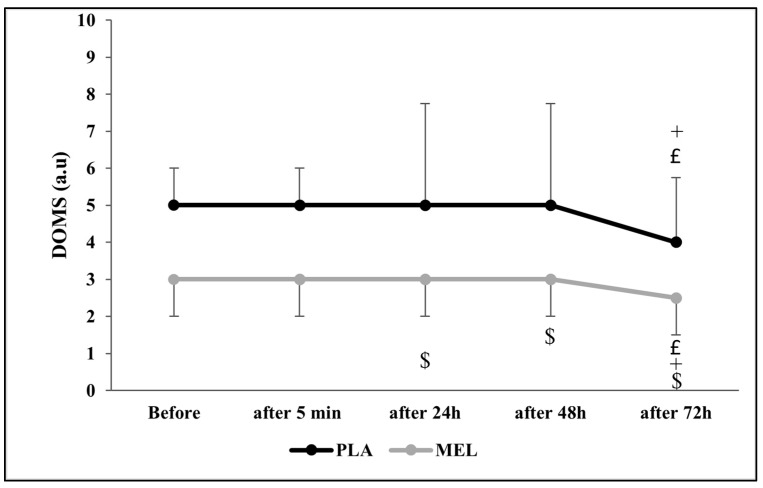
Delayed onset muscle soreness (DOMS) scores recorded before, 5 min, 24 h, 48 h, and 72 h after the 5 m shuttle run test in PLA and MEL condition (n = 12). Values are presented as median (interquartile range); £ *p* < 0.05: significant difference in comparison with values measured 24 h after the 5mSRT within each condition. + *p* < 0.05: significant difference in comparison with values measured 48 h after the 5mSRT within each condition. $ *p* < 0.05 significant difference between PLA and MEL at time pairwise comparisons. MEL: melatonin; PLA: placebo; a.u: arbitrary unit.

The Friedman test revealed a significant effect of time on DOMS across conditions (Chi^2^ = 43.294; N = 12; df = 9; *p* < 0.001). The Wilcoxon test showed a significant effect within the PLA condition, with PLA 24 h post-exercise resulting in higher DOMS values than PLA at 72 h and MEL 24 h after testing (Z = −2.266; r = 0.654; *p =* 0.023 and Z = −2.322; r = 0.670; *p =* 0.020, respectively). Furthermore, PLA 48 h after the 5mSRT elicited higher values PLA 24 h and MEL 48 h after exercise (Z = −2.232; r = 0.644; *p =* 0.026 and Z = −2.290; r = 0.661; *p =* 0.022, respectively). In addition, MEL after 72 h resulted in lower values in comparison with PLA 72 h (Z = −2.733; r = 0.788; *p =* 0.006), MEL 24 h (Z = −1.980; r = 0.571; *p =* 0.048), and MEL 72 h (Z = −2.121; r = 0.612; *p =* 0.034) after the test (Figure 4).

## 4. Discussion

The present study investigated the effects of the nocturnal ingestion of 6 mg of melatonin on sleep quality and quantity, biochemical, physiological, and perceptive responses, and physical performance in trained males. The main findings were that MEL did not modify any sleep variables in the night after ingestion. However, MEL showed potential ergogenic effects in the following day performance-related variables during the 5mSRT. The improved physical performance was achieved with a lower HR_peak_ and no impact on RPE. After exercise, MEL did not reduce serum muscle damage or inflammation markers but induced a better recovery process for the 72 h after exercise with better PRS and DOMS levels. In summary, while 6 mg of nocturnal melatonin ingestion did not impact sleep variables, post-exercise muscle damage, or inflammation markers, it demonstrated potential ergogenic benefits and significantly improved recovery indicators over the 72 h following intense exercise.

The current investigation showed that MEL ingestion did not significantly affect sleep variables (Table 2). Similar results were previously reported by Atkinson et al. [39] who showed no meaningful effect of 5 mg of MEL intake 30 min before bedtime on sleep variables. However, other previous investigations have shown that the nighttime ingestion of 10 mg [18] and 8 mg MEL [19] improved subjective sleep quality. Specifically, Cheikh et al. [18] reported that MEL supplementation led to increases in total sleep time, sleep efficiency, stage-3 sleep, and rapid-eye-movement sleep, and reduced sleep onset-latency, total time of nocturnal awakenings after sleep onset, stage-1 sleep, and stage-2 sleep, while no effect was recorded for the time in bed. This divergence observed between the studies could be attributed to the difference in terms of the dose and time of MEL administration as well as the level of sleep disruption, the age [20], and the chronotype of participants [18], which may limit the efficacy of MEL administration in the present study as compared to previous ones. Collectively, the current study found no significant effects of MEL ingestion on sleep variables. However, discrepancies with other studies reporting improved subjective sleep quality at higher MEL doses highlight the potential influence of factors such as dosage, timing of administration, participant characteristics (e.g., sleep disruption, age, and chronotype), and study design, which may have limited the efficacy of MEL in the present investigation. Based on the evidence presented, higher doses of melatonin, such as 8–10 mg, appear to provide more consistent benefits for sleep quality, including improvements in total sleep time, sleep efficiency, and reduced sleep onset latency. Therefore, it is recommended to consider these higher doses for individuals seeking melatonin’s sleep-enhancing effects, while also tailoring the timing of administration and accounting for individual factors like age, chronotype, and baseline sleep disruptions to maximize efficacy.

Regarding physical performance and HR responses, the present study reported improved physical performance (i.e., TD, FI, and PD) and reduced HR_peak_ values during the 5mSRT. Notably, out of 12 participants, 10 obtained higher TD with MEL condition, 10 exhibited reduced FI, and 10 showed a lower PD between sprints in the 5mSRT. This indicated that most of the participants responded positively to MEL, although there was some inter-individual variability in response to this supplementation. The results from previous investigations are conflicting about the effects of MEL ingestion. In fact, a body of evidence has confirmed the ergogenic potential of MEL to improve short-term and endurance performances [2,12,18,19]. These later studies concluded that 10 mg of nocturnal MEL ingested after exhaustive late evening exercise (i.e., after performing RAST) induced a positive effect on the performance of the same test on the following morning, by increasing peak power and mean power, and decreasing the total time and the FI in healthy male adolescent athletes [2]. Likewise, Cheikh et al. [18] showed that morning performance assessed by the YYIRT-1 and the five-jump test was enhanced following 10 mg of MEL ingested after the endurance test and 15 min before bedtime. Additional data from a study by Paryab et al. [12] reported that 6 mg of nocturnal MEL was effective in improving short-term performances after 24 h of sleep deprivation. Recently, 8 mg of MEL nocturnal intake was effective in improving handgrip, squat jump, Wingate peak, and mean powers in 12 professional soccer players [19]. On the contrary, previous investigations demonstrated that MEL ingestion had no significant effect on physical performance either after nocturnal [4,10,39] or diurnal [7,14,21,24,40] administrations. Methodological inconsistencies between studies, such as the dose, time of ingestion, exercise type used, and physical fitness level of participants, could be potential explanations for these disparities in findings [19]. In addition, to the current authors’ knowledge, only a study by Atkinson et al. [21] reported a slightly significant decrease in HR values of 6 to 9 beats/min, after daytime ingestion of 5 mg of MEL, whereas, other studies did not detect any difference between MEL and PLA conditions [4,18,40,41,42,43]. The HR decrease following MEL ingestion was linked to deleting sympathetic’ suppression [44] and catecholamine levels reduction [45]. Regarding perceived exertion, the RPE scores recorded after the 5mSRT were not affected by MEL ingestion. This contrasts with Ghattassi et al. [19], where 8 mg of oral MEL intake before sleep, decreased the RPE score after the Wingate anaerobic test on the following day. However, Souissi et al. [43] found that RPE increased under 6 mg of MEL compared to PLA condition. On the other hand, our results align with previous studies [4,10,21,39,40,41] that linked the lack of difference between conditions to the intensity of tests used. Collectively, all this information suggests that MEL may be ergogenic when ingested the night before testing, potentially enhancing physical performance and reducing HR responses without affecting fatigue.

Despite previous studies showing that the 5mSRT induces clear signs of muscle damage (pre-to-post changes in serum CK, LDH, ASAT, ALAT) and inflammation (CRP) markers [46], the results from the current study showed that the same exercise did not affect the serum concentration of ASAT, ALAT, and CRP, while it increased only CK and LDH in both conditions. In any case, MEL ingestion did not affect serum concentrations of these serum makers when compared to PLA (Table 1). These data suggest that acute nocturnal MEL ingestion did not provide a protective role against exercise-induced muscle damage and inflammation, at least in a dose of 6 mg. According to the literature, numerous studies were conducted to assess the acute [2,4,47] or chronic effect [20,25,48,49] of MEL administration on various cellular damage and/or inflammation markers. Contrary to our results, those authors concluded that MEL, intake either before bedtime or before exercise, protected athletes from possible increases in those biomarkers. Whereas our findings were similar to those reported by a study by Souissi et al. [50] which showed that 6 mg of MEL consumed before exercising did not elicit any significant effect on LDH, ASAT, ALAT, and CRP in moderately trained students. Furthermore, the same dose of MEL 50 min prior to performing a prolonged, submaximal and continuous exercise (i.e., time to exhaustion trial) did not buffer the increase in muscle creatine kinase (CK-NAK) and LDH in moderately active men [51]. It is possible that the type of exercise used in this study, which was highly intense but relatively short in time contributed to low levels of muscle damage and inflammation, at least in comparison to the muscle damage reported in longer exercise events such as marathons [52] or half-ironman [53]. Therefore, the study of the effect of MEL intake in longer endurance-based events is required to ascertain if MEL is more effective in those exercise activities with higher levels of muscle damage.

Our study revealed a positive effect of MEL on the levels of PRS and DOMS compared to PLA which persisted up to 72 h after exercise. Such positive effects can be explained by the nociceptive, analgesic, anti-inflammatory, and antioxidant potential [54]. To our current knowledge, no previous studies have examined the effect of MEL supplementation on PRS; however, some reports [20,49] concluded that daily 5 mg of nocturnal MEL ingested during a 6-day training camp could have beneficial effects on athletes’ recovery by reducing oxidative stress and muscle damage during exercise. In contrast, others did not find any significant effects of 6 mg of diurnal MEL intake before the training sessions [10,24]. In summary, our study demonstrated that MEL supplementation positively influenced PRS levels and maintained this effect for 72 h post-exercise. While no prior studies have specifically explored MEL’s impact on PRS, existing evidence suggests that nocturnal MEL supplementation may enhance recovery by mitigating oxidative stress and muscle damage, although findings regarding diurnal MEL intake remain inconclusive.

### Strength, Limitations, and Perspectives

This study is among the few that have evaluated the acute effects of nocturnal melatonin ingestion on sleep quality and next-day performance outcomes during periods of intensive exercise. The findings may offer valuable insights for coaches and trainers aiming to enhance recovery after strenuous training sessions or optimize preparation for competition days. However, some limitations should be acknowledged. First, the investigation focused only on acute effects of MEL administration and leaving the potential benefits of chronic intake unexplored. In addition, we did not investigate the effect of MEL on delayed biochemical responses, up to 72 after exercise. It is common to observe that serum concentrations of muscle damage markers increase several hours after exercise, raising the possibility that MEL may have influenced these variables beyond the post-exercise measurement carried out in this study. Third, we did not measure complementary variables such as core body temperature, serum MEL, and cortisol levels which could have helped us to understand the mechanism(s) behind MEL’s ergogenic properties found in this study. The findings of this study are specific to a 6 mg dose of nocturnal MEL supplementation. It is still possible that the ingestion of a higher dose of MEL would have produced a greater benefit in performance and better values of perceived recovery and muscle pain. Further studies exploring various doses of MEL are warranted to evaluate the potential dose–response relationship for its effects on performance and recovery following intense exercise, particularly to detect the minimal effective dose of MEL with a potential ergogenic benefit. This study was carried out with a relatively small sample of trained males. We utilized a randomized, crossover, or counterbalanced design and included a placebo condition to isolate the performance effects of MEL. Additionally, we implemented pre-experimental standardizations for sleep and exercise habits and maintained consistent exercise testing protocols. However, some results may still be influenced by natural day-to-day variations in exercise performance. To confirm the potential benefits found in this study, further investigations should be carried out, using a large sample size with a gender comparison and the inclusion of high-performance athletes to generalize the effects of MEL on athletic performance. Lastly, the effects of MEL may be more pronounced in individuals with sleep disorders, a possibility that warrants further investigation, as the participants in this study did not report any pre-existing sleep issues.

## 5. Conclusions

The present study showed that ingesting 6 mg of melatonin at night positively influenced some physical performance variables during highly intense and repetitive exercise the following day, even with no improvements in sleep parameters. Specifically, melatonin intake enhanced total distance and fatigue index and reduced the percentage decrement between sprints in the 5 m shuttle test. The improved physical performance during this test was achieved with lower values of peak heart rate and no impact on RPE. Additionally, although no improvements were observed in biochemical markers of muscle damage and inflammation after exercise, melatonin ingestion enhanced perceived recovery for up to 72 h post-exercise, as indicated by higher PRS scores and lower DOMS values compared to the placebo. These outcomes suggest that nocturnal MEL ingestion could serve as a potential supplementation strategy to enhance high-intensity physical performance the day after in trained males. From a practical standpoint, this offers a simple, legal and accessible option for athletes or trained individuals to improve recovery and next-day performance, particularly in contexts where performance and recovery the following day are important, such as after intense evening training or competition. However, more research is needed to determine the optimal dose and timing of nocturnal MEL ingestion to maximize its benefits on exercise performance. Since the number of studies on the effects of melatonin on exercise performance is limited, further crossover trials with larger sample sizes are needed to strengthen the inferences regarding its potential ergogenic benefits in exercise and sports contexts. Last, further studies should investigate the mechanisms underlying the enhanced self-perceived recovery in the days following exercise with melatonin to better understand the potential pathways involved in melatonin-induced recovery improvement.

## Figures and Tables

**Table 1:** Characteristics of the participants of this study (N = 12).

Variable (Units)	Mean ± SD
Age (yrs)	21.92 ± 2.84
Body weight (kg)	77.00 ± 4.24
Height (cm)	1.80 ± 0.05
Body mass index (kg/m^2^)	22.57 ± 2.57
Training volume (hours/week)	4 ± 2
Training experience (yrs)	5.33 ± 6.67

SD: Standard deviation.

**Table 2:** Sleep parameters recorded in both PLA and MEL conditions (n = 12).

	PLA	MEL
In bedtime (hh:min)	22:35 ± 00:51	22:33 ± 00:28
Out bedtime (hh:min)	06:54 (00:09)	06:55 (00:11)
Sleep latency (min)	75.67 ± 48.68	72.42 ± 50.58
Sleep efficiency (%)	60.39 ± 16.40	66.16 ± 4.64
Total time in bed (min)	496.08 ± 56.97	503.00 ± 34.59
Total sleep time (min)	325.00 (98.00)	330.50 (56.00)

PLA: placebo; MEL: melatonin. Data are presented as mean ± standard deviation for variables with a normal distribution and as median (interquartile range) for variables with non-normal distribution.

**Table 3:** Physical performance, peak heart rate (HRpeak), and rating of perceived exertion (RPE) recorded in PLA and MEL conditions (n = 12).

	PLA	MEL
Total distance (m)	625.0 ± 81.0	747.5 ± 109.0 *
Best distance (m)	122.5 ± 26.0	130.0 ± 23.0
Fatigue index (%)	15.75 ± 9.10	4.29 ± 3.04 *
Percentage decrement (%)	14.12 ± 4.84	4.09 ± 3.18 *
HRpeak (beat/min)	188 ± 7	182 ± 3 *
RPE (a.u)	6.79 ± 1.22	6.27 ± 1.34

* *p* < 0.05: difference in comparison with values measured in the placebo condition. PLA: placebo; MEL: melatonin; RPE: rating of perceived exertion; a.u: arbitrary unit. Data are presented as mean ± standard deviation for variables with a normal distribution and as median (interquartile range) for variables with non-normal distribution.

**Table 4:** Biochemical parameters recorded before and 5 min after the 5mSRT in PLA and MEL conditions (n = 12).

	PLA		MEL	
	Before	After	Before	After
CK (IU/L)	434.0 (513.0)	467.0 (506.0) *	461.0 (407.0)	525.0 (429.0) *
LDH (IU/L)	201.5 (67)	234.5 (70.0) *	204.5 (63.0)	233.0 (71.0) *
ASAT (IU/L)	31.15 ± 14.56	36.25 ± 14.94	33.5 ± 13.89	31.33 ± 13.29
ALAT (IU/L)	25.0 ± 9.24	27.42 ± 10.27	23.25 ± 9.76	26.25 ± 10.88
CRP (mg/L)	10.0 (5.0)	12.1 (5.7) *	10.75 (12.5)	11.85 (15.7)

* *p* < 0.05: significant difference in comparison with values measured before the 5mSRT within each condition. PLA: placebo; MEL: melatonin; CK: creatine kinase; LDH: lactate dehydrogenase; ASAT: aspartate aminotransferase; ALAT: alanine aminotransferase; CRP: C-creatine protein. Data are presented as mean ± standard deviation for variables with a normal distribution and as median (interquartile range) for variables with non-normal distribution.

## Data Availability

The datasets presented in this article are not readily available [the data are part of an ongoing study]. Requests to access the datasets should be directed to the corresponding author.

## Abbreviations

ASAT	Aspartate aminotransferase
ALAT	Alanine aminotransferase
CRP	C-reactive protein
DOMS	Delayed onset muscle recovery
FI	Fatigue index
HR_peak_	Peak heart rate
PD	Percentage decrement
5mSRT	5 m shuttle run test
CK	Creatine kinase
LDH	Lactate dehydrogenase
PRS	Perceived recovery status
MEL	Melatonin
PLA	Placebo
RPE	Rating of perceived exertion
