# Correction: Mahdi et al. Melatonin Supplementation Enhances Next-Day High-Intensity Exercise Performance and Recovery in Trained Males: A Placebo-Controlled Crossover Study. *Sports* 2025, *13*, 190

**DOI:** 10.3390/sports14050176

**Published:** 2026-04-28

**Authors:** Nourhène Mahdi, Slaheddine Delleli, Arwa Jebabli, Khouloud Ben Maaoui, Juan Del Coso, Hamdi Chtourou, Luca Paolo Ardigò, Ibrahim Ouergui

**Affiliations:** 1 High Institute of Sport and Physical Education of Sfax, University of Sfax, Sfax 3000, Tunisia; nourhene648@gmail.com (N.M.); sdelleli2018@gmail.com (S.D.); jebabliarwa@gmail.com (A.J.); benmaaouikhouloud88@gmail.com (K.B.M.); h_chtourou@yahoo.fr (H.C.); 2 Research Laboratory, Education, Motricity, Sport and Health, EM2S, LR19JS01, University of Sfax, Sfax 3000, Tunisia; 3 Physical Activity, Sport and Health, Research Unit, UR18JS01, National Sport Observatory, Tunis 1003, Tunisia; 4 Centre for Sport Studies, Rey Juan Carlos University, 28943 Fuenlabrada, Spain; juan.delcoso@urjc.es; 5 Department of Teacher Education, NLA University College, 0166 Oslo, Norway; 6 High Institute of Sport and Physical Education of Kef, University of Jendouba, Kef 7100, Tunisia; 7 Research Unit: Sport Sciences, Health and Movement, UR22JS01, University of Jendouba, Kef 7100, Tunisia

In the published paper [1], there are some details that need to be corrected:

In Section 2.1, paragraph 1, the eligibility criteria originally described participants as “physically active (≥3 h of exercise training/week).” Since this study specifically involved trained athletes, it was changed to “trained athletes” to better reflect the actual sample. Similarly, the “physically active individuals” in the Conclusions section also needs to be modified to “trained individuals”. Accordingly, in the recruitment flow (Figure 1), there was a mistake in Figure 1 as published in the original version. The statement of “Physically active men” needs to be updated to “trained males”. The corrected Figure 1 appears below.

In Section 2.3.1, paragraph 1, the fatigue index (FI) for the 5mSRT should be divided by 2.

Previous formula: Fatigue index (FI) (%) = [((Shuttle 1 + Shuttle 2) − (Shuttle 5 + Shuttle 6))/(Shuttle 1 + Shuttle 2)] × 100.

The correct version: 

Fatigue index (FI) (%) = {[((Shuttle 1 + Shuttle 2)/2) − ((Shuttle 5 + Shuttle 6)/2)]/((Shuttle 1 + Shuttle 2)/2)} × 100

In the section on Physical and Physiological Parameters (In Section 3.2, paragraph 1), the reported value Z = −2824 was a typographical mistake. The correct value is Z = −2.824.

The authors state that the scientific conclusions are unaffected. This correction was approved by the Academic Editor. The original publication has also been updated.

## Figures and Tables

**Figure 1:**
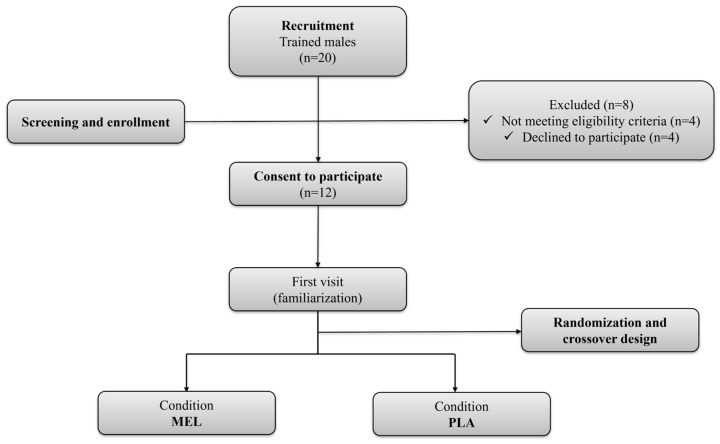
Flowchart of participants. MEL: melatonin; PLA: placebo.
